# *trans*-Diaqua­bis(6-methoxycarbonyl­pyridazine-3-carboxyl­ato-κ^2^ *N*,*O*)zinc(II) dihydrate

**DOI:** 10.1107/S1600536809017115

**Published:** 2009-05-14

**Authors:** Wojciech Starosta, Janusz Leciejewicz

**Affiliations:** a Institute of Nuclear Chemistry and Technology, ul. Dorodna 16, 03-195 Warszawa, Poland

## Abstract

In the title centrosymmetric complex, [Zn(C_7_H_5_N_2_O_4_)_2_(H_2_O)_2_]·2H_2_O, the Zn^II^ ion is coordinated in a *trans* mode by two symmetry-related bis-chelating 6-methoxycarbonyl­pyridazine-3-carboxyl­ate ligands *via* N and O atoms, and by two aqua ligand O atoms in axial positions, in a slightly distorted octa­hedral environment. In the crystal structure, complex mol­ecules are linked by inter­molecular O—H⋯O hydrogen bonds between coordinated and solvent water mol­ecules and carboxyl­ate O atoms, forming mol­ecular ribbons propagating along the *a* axis.

## Related literature

For the crystal structures of two zinc complexes with pyridazine-3-carboxyl­ate and water ligands, see: Gryz *et al.* (2003 ▶, 2004 ▶). For a centrosymmetric dimeric zinc(II) complex with pyridazine-3,6-dicarboxyl­ate and water ligands, see: Gryz *et al.* (2006 ▶). For modifications of pyridazine-3,6-dicarboxylic acid, see: Starosta & Leciejewicz (2004 ▶); Sueur *et al.* (1987 ▶).
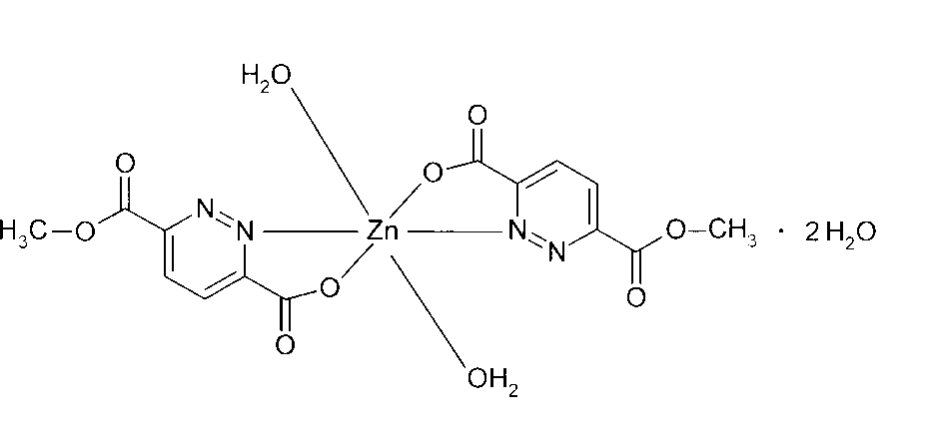


## Experimental

### 

#### Crystal data

[Zn(C_7_H_5_N_2_O_4_)_2_(H_2_O)_2_]·2H_2_O
*M* *_r_* = 499.69
Triclinic, 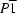

*a* = 6.3678 (13) Å
*b* = 8.3178 (17) Å
*c* = 9.7717 (19) Å
α = 102.99 (3)°
β = 108.90 (3)°
γ = 94.97 (3)°
*V* = 469.93 (17) Å^3^
*Z* = 1
Mo *K*α radiation
μ = 1.38 mm^−1^
*T* = 293 K
0.31 × 0.25 × 0.05 mm

#### Data collection

Kuma KM4 four-circle diffractometer
Absorption correction: analytical (*CrysAlis RED*; Oxford Diffraction, 2008 ▶) *T*_min_ = 0.699, *T*_max_ = 0.942
2787 measured reflections
2595 independent reflections
2235 reflections with *I* > 2σ(*I*)
*R*_int_ = 0.022
3 standard reflections every 200 reflections intensity decay: none

#### Refinement

*R*[*F* ^2^ > 2σ(*F* ^2^)] = 0.035
*wR*(*F* ^2^) = 0.101
*S* = 1.07
2595 reflections
159 parameters
H atoms treated by a mixture of independent and constrained refinement
Δρ_max_ = 0.65 e Å^−3^
Δρ_min_ = −0.75 e Å^−3^

### 

Data collection: *KM-4 Software* (Kuma, 1996 ▶); cell refinement: *KM-4 Software*; data reduction: *DATAPROC* (Kuma, 2001 ▶); program(s) used to solve structure: *SHELXS97* (Sheldrick, 2008 ▶); program(s) used to refine structure: *SHELXL97* (Sheldrick, 2008 ▶); molecular graphics: *SHELXTL* (Sheldrick, 2008 ▶); software used to prepare material for publication: *SHELXL97*.

## Supplementary Material

Crystal structure: contains datablocks I, global. DOI: 10.1107/S1600536809017115/lh2816sup1.cif

Structure factors: contains datablocks I. DOI: 10.1107/S1600536809017115/lh2816Isup2.hkl

Additional supplementary materials:  crystallographic information; 3D view; checkCIF report

## Figures and Tables

**Table 1:** Hydrogen-bond geometry (Å, °)

*D*—H⋯*A*	*D*—H	H⋯*A*	*D*⋯*A*	*D*—H⋯*A*
O2—H22⋯O11^i^	0.93 (4)	1.99 (4)	2.874 (3)	158 (4)
O2—H21⋯O21^ii^	0.72 (4)	2.23 (4)	2.940 (3)	168 (4)
O1—H12⋯O12^i^	0.84 (3)	1.86 (3)	2.695 (2)	174 (3)
O1—H11⋯O2	0.90 (4)	1.89 (4)	2.720 (2)	152 (3)

Symmetry codes: (i) 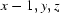; (ii) 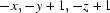.

**Fig. 1.**
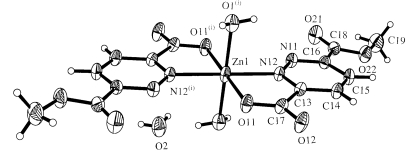
The molecular structure of (I) with atom labelling scheme and 50% probability displacement ellipsoids. Symmetry code:(i) -x + 1, -y + 1, -z + 1. The symmetry related solvent water molecule is not shown.

**Fig. 2.**
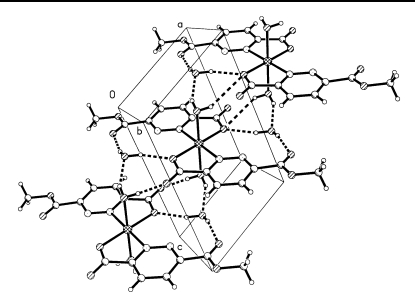
Part of the crystal structure with hydrogen bonds shown as dashed lines.

## supplementary crystallographic information

### Comment

In the molecluar structure of the title compound (I) (Fig.1) the Zn^II^ ion, which is located on a center of symmetry, is coordinated in trans mode by two, symmetry related, bis chelating ligand molecules through their N,O bonding atoms. Two water O atoms in axial positions complete the number of coordinated atoms to six. The coordination geometry is slightly distorted octahedral. Bond distances and bond angles are close to those reported for two zinc complexes with pyridazine-3-carboxylate and water ligands (Gryz *et al.*, 2003, 2004), a complex with pyridazine-3,6-dicarboxylate and water ligands (Gryz *et al.*, 2006) and for both modifications of pyridazine-3,6-dicarboxylic acid (Sueur *et al.*, 1987; Starosta & Leciejewicz, 2004). The ligand molecules and the Zn^II^ ion are almost coplanar [r.m.s. 0.0074 Å]. The carboxylic C12/O11/O12 and the carboxymethyl C18/O21/O22/C19 groups make dihedral angles with the pyridazine ring of 3.0 (2) and 6.8 (1)°, respectively. In the crystal structure complex molecules are linked by hydrogen bonds to form molecular ribbons (Fig. 2). The relevant hydrogen-bond parameters are listed in Table 1.

### Experimental

Hot aqueous solutions containing 2 mmol of 6-carboxymethylpyridazine-3-carboxylic acid and 1 mmol of zinc(II) acetate tetrahydrate, respectively, were mixed and boiled for two hours with constant stirring and then left to crystallize at room temperature. After few days, well formed colorless single crystals were found in the mother liquid in the mass of polycrystalline material. The crystals were washed with cold ethanol and dried in air.

### Refinement

H atoms bonded to C atoms were placed in calculated positions with C—H = 0.93 and 0.96 Å and included in a riding-model approximation with U_iso_(H) = 1.2U_eq_(C) or 1.5U_eq_(C) for methyl atoms. Water H atoms were located in difference Fourier maps and were refined isotropically.

### Crystal data

**Table:**

[Zn(C_7_H_5_N_2_O_4_)_2_(H_2_O)_2_]·2H_2_O	*Z* = 1
*M**_r_* = 499.69	*F*(000) = 256
Triclinic, *P*1	*D*_x_ = 1.766 Mg m^−^^3^
Hall symbol: -P 1	Mo *K*α radiation, λ = 0.71073 Å
*a* = 6.3678 (13) Å	Cell parameters from 25 reflections
*b* = 8.3178 (17) Å	θ = 6–15°
*c* = 9.7717 (19) Å	µ = 1.38 mm^−^^1^
α = 102.99 (3)°	*T* = 293 K
β = 108.90 (3)°	Plate, colourless
γ = 94.97 (3)°	0.31 × 0.25 × 0.05 mm
*V* = 469.93 (17) Å^3^	

### Data collection

**Table:**

Kuma KM4 four-circle diffractometer	2235 reflections with *I* > 2σ(*I*)
Radiation source: fine-focus sealed tube	*R*_int_ = 0.022
graphite	θ_max_ = 30.1°, θ_min_ = 2.3°
profile data from ω/2θ scans	*h* = 0→8
Absorption correction: analytical (*CrysAlis RED*; Oxford Diffraction, 2008)	*k* = −8→11
*T*_min_ = 0.699, *T*_max_ = 0.942	*l* = −13→13
2787 measured reflections	3 standard reflections every 200 reflections
2595 independent reflections	intensity decay: 0.0%

### Refinement

**Table:**

Refinement on *F*^2^	Primary atom site location: structure-invariant direct methods
Least-squares matrix: full	Secondary atom site location: difference Fourier map
*R*[*F*^2^ > 2σ(*F*^2^)] = 0.035	Hydrogen site location: inferred from neighbouring sites
*wR*(*F*^2^) = 0.101	H atoms treated by a mixture of independent and constrained refinement
*S* = 1.07	*w* = 1/[σ^2^(*F*_o_^2^) + (0.0761*P*)^2^ + 0.0253*P*] where *P* = (*F*_o_^2^ + 2*F*_c_^2^)/3
2595 reflections	(Δ/σ)_max_ = 0.002
159 parameters	Δρ_max_ = 0.65 e Å^−^^3^
0 restraints	Δρ_min_ = −0.75 e Å^−^^3^

### Special details

**Table:**

Geometry. All e.s.d.'s (except the e.s.d. in the dihedral angle between two l.s. planes) are estimated using the full covariance matrix. The cell e.s.d.'s are taken into account individually in the estimation of e.s.d.'s in distances, angles and torsion angles; correlations between e.s.d.'s in cell parameters are only used when they are defined by crystal symmetry. An approximate (isotropic) treatment of cell e.s.d.'s is used for estimating e.s.d.'s involving l.s. planes.
Refinement. Refinement of *F*^2^ against ALL reflections. The weighted *R*-factor *wR* and goodness of fit *S* are based on *F*^2^, conventional *R*-factors *R* are based on *F*, with *F* set to zero for negative *F*^2^. The threshold expression of *F*^2^ > σ(*F*^2^) is used only for calculating *R*-factors(gt) *etc*. and is not relevant to the choice of reflections for refinement. *R*-factors based on *F*^2^ are statistically about twice as large as those based on *F*, and *R*- factors based on ALL data will be even larger.

### Fractional atomic coordinates and isotropic or equivalent isotropic displacement parameters (Å^2^)

**Table:**

	*x*	*y*	*z*	*U*_iso_*/*U*_eq_	
Zn1	0.5000	0.5000	0.5000	0.02700 (11)	
O11	0.8125 (2)	0.43757 (16)	0.58939 (15)	0.0313 (3)	
O12	1.0055 (2)	0.22915 (17)	0.57098 (18)	0.0371 (3)	
N11	0.2403 (2)	0.15325 (19)	0.29574 (15)	0.0255 (3)	
N12	0.4382 (2)	0.24189 (17)	0.39017 (15)	0.0235 (3)	
C17	0.8332 (3)	0.2900 (2)	0.53776 (18)	0.0253 (3)	
C13	0.6217 (3)	0.1734 (2)	0.42383 (17)	0.0226 (3)	
C14	0.6202 (3)	0.0068 (2)	0.36089 (19)	0.0278 (3)	
H14	0.7511	−0.0393	0.3832	0.033*	
O1	0.4013 (2)	0.44171 (18)	0.67752 (15)	0.0326 (3)	
H11	0.382 (6)	0.535 (5)	0.738 (4)	0.060 (9)*	
O21	−0.1599 (2)	−0.0300 (2)	0.10069 (19)	0.0436 (3)	
C16	0.2308 (3)	−0.0079 (2)	0.23612 (17)	0.0242 (3)	
C18	−0.0011 (3)	−0.0981 (2)	0.13509 (17)	0.0272 (3)	
C15	0.4172 (3)	−0.0876 (2)	0.26422 (19)	0.0297 (3)	
H15	0.4048	−0.2008	0.2191	0.036*	
O22	−0.0035 (2)	−0.25921 (17)	0.09206 (16)	0.0378 (3)	
C19	−0.2178 (4)	−0.3623 (3)	−0.0046 (3)	0.0479 (5)	
H191	−0.3354	−0.2966	−0.0089	0.072*	
H192	−0.2459	−0.4546	0.0346	0.072*	
H193	−0.2146	−0.4041	−0.1036	0.072*	
O2	0.1965 (3)	0.6755 (3)	0.8035 (2)	0.0493 (4)	
H12	0.274 (5)	0.379 (4)	0.639 (3)	0.043 (7)*	
H21	0.207 (6)	0.765 (5)	0.830 (4)	0.061 (11)*	
H22	0.054 (7)	0.622 (6)	0.738 (4)	0.074 (10)*	

### Atomic displacement parameters (Å^2^)

**Table:**

	*U*^11^	*U*^22^	*U*^33^	*U*^12^	*U*^13^	*U*^23^
Zn1	0.01928 (14)	0.01626 (16)	0.03827 (16)	0.00521 (9)	0.00402 (10)	0.00167 (10)
O11	0.0207 (5)	0.0216 (6)	0.0435 (6)	0.0055 (4)	0.0047 (5)	0.0023 (5)
O12	0.0205 (5)	0.0257 (7)	0.0593 (8)	0.0086 (5)	0.0064 (5)	0.0095 (6)
N11	0.0200 (6)	0.0217 (6)	0.0317 (6)	0.0038 (5)	0.0065 (5)	0.0048 (5)
N12	0.0199 (6)	0.0177 (6)	0.0308 (6)	0.0042 (5)	0.0073 (5)	0.0045 (5)
C17	0.0190 (6)	0.0219 (7)	0.0346 (7)	0.0040 (5)	0.0080 (5)	0.0088 (6)
C13	0.0206 (6)	0.0193 (7)	0.0288 (6)	0.0045 (5)	0.0092 (5)	0.0071 (5)
C14	0.0230 (7)	0.0223 (8)	0.0373 (8)	0.0080 (6)	0.0098 (6)	0.0065 (6)
O1	0.0249 (6)	0.0286 (6)	0.0404 (6)	0.0026 (5)	0.0095 (5)	0.0053 (5)
O21	0.0268 (6)	0.0339 (8)	0.0563 (8)	0.0047 (5)	0.0021 (6)	0.0040 (6)
C16	0.0237 (7)	0.0200 (7)	0.0265 (6)	0.0024 (5)	0.0072 (5)	0.0043 (5)
C18	0.0271 (7)	0.0233 (8)	0.0271 (6)	0.0004 (6)	0.0072 (6)	0.0038 (5)
C15	0.0312 (8)	0.0181 (7)	0.0369 (8)	0.0060 (6)	0.0113 (6)	0.0022 (6)
O22	0.0329 (6)	0.0231 (6)	0.0429 (7)	−0.0004 (5)	0.0017 (5)	−0.0002 (5)
C19	0.0403 (10)	0.0297 (10)	0.0535 (11)	−0.0072 (9)	0.0010 (9)	−0.0003 (8)
O2	0.0391 (8)	0.0403 (10)	0.0611 (10)	0.0129 (7)	0.0139 (7)	0.0023 (8)

### Geometric parameters (Å, °)

**Table:**

Zn1—O11	2.0617 (13)	C14—H14	0.9300
Zn1—O11^i^	2.0617 (13)	O1—H11	0.90 (4)
Zn1—N12^i^	2.1118 (15)	O1—H12	0.84 (3)
Zn1—N12	2.1118 (15)	O21—C18	1.192 (2)
Zn1—O1^i^	2.1612 (14)	C16—C15	1.390 (2)
Zn1—O1	2.1612 (14)	C16—C18	1.500 (2)
O11—C17	1.253 (2)	C18—O22	1.309 (2)
O12—C17	1.227 (2)	C15—H15	0.9300
N11—C16	1.324 (2)	O22—C19	1.445 (2)
N11—N12	1.3277 (19)	C19—H191	0.9600
N12—C13	1.323 (2)	C19—H192	0.9600
C17—C13	1.520 (2)	C19—H193	0.9600
C13—C14	1.382 (2)	O2—H21	0.72 (4)
C14—C15	1.371 (2)	O2—H22	0.93 (4)
			
O11—Zn1—O11^i^	180.0	C14—C13—C17	122.46 (14)
O11—Zn1—N12^i^	101.21 (6)	C15—C14—C13	117.11 (16)
O11^i^—Zn1—N12^i^	78.79 (6)	C15—C14—H14	121.4
O11—Zn1—N12	78.79 (6)	C13—C14—H14	121.4
O11^i^—Zn1—N12	101.21 (6)	Zn1—O1—H11	111 (2)
N12^i^—Zn1—N12	180.0	Zn1—O1—H12	108.5 (18)
O11—Zn1—O1^i^	89.08 (6)	H11—O1—H12	106 (3)
O11^i^—Zn1—O1^i^	90.91 (6)	N11—C16—C15	123.58 (15)
N12^i^—Zn1—O1^i^	89.79 (6)	N11—C16—C18	113.77 (15)
N12—Zn1—O1^i^	90.20 (6)	C15—C16—C18	122.66 (15)
O11—Zn1—O1	90.92 (6)	O21—C18—O22	125.50 (16)
O11^i^—Zn1—O1	89.08 (6)	O21—C18—C16	123.73 (16)
N12^i^—Zn1—O1	90.21 (6)	O22—C18—C16	110.77 (15)
N12—Zn1—O1	89.80 (6)	C14—C15—C16	117.52 (16)
O1^i^—Zn1—O1	180.00 (6)	C14—C15—H15	121.2
C17—O11—Zn1	116.33 (11)	C16—C15—H15	121.2
C16—N11—N12	117.85 (14)	C18—O22—C19	116.76 (16)
C13—N12—N11	121.74 (14)	O22—C19—H191	109.5
C13—N12—Zn1	112.69 (11)	O22—C19—H192	109.5
N11—N12—Zn1	125.56 (11)	H191—C19—H192	109.5
O12—C17—O11	126.85 (16)	O22—C19—H193	109.5
O12—C17—C13	116.54 (15)	H191—C19—H193	109.5
O11—C17—C13	116.61 (14)	H192—C19—H193	109.5
N12—C13—C14	122.16 (15)	H21—O2—H22	114 (4)
N12—C13—C17	115.37 (14)		

Symmetry codes: (i) −*x*+1, −*y*+1, −*z*+1.

### Hydrogen-bond geometry (Å, °)

**Table:**

*D*—H···*A*	*D*—H	H···*A*	*D*···*A*	*D*—H···*A*
O2—H22···O11^ii^	0.93 (4)	1.99 (4)	2.874 (3)	158 (4)
O2—H21···O21^iii^	0.72 (4)	2.23 (4)	2.940 (3)	168 (4)
O1—H12···O12^ii^	0.84 (3)	1.86 (3)	2.695 (2)	174 (3)
O1—H11···O2	0.90 (4)	1.89 (4)	2.720 (2)	152 (3)

Symmetry codes: (ii) *x*−1, *y*, *z*; (iii) −*x*, −*y*+1, −*z*+1.
